# Visual Field Loss Morphology in High- and Normal-Tension Glaucoma

**DOI:** 10.1155/2012/327326

**Published:** 2012-02-08

**Authors:** Michele Iester, Fabio De Feo, Gordon R. Douglas

**Affiliations:** ^1^Department of Ophthalmology, University of British Columbia, Vancouver, BC, Canada V5Z 3N9; ^2^Anatomical-Clinical Laboratory for Functional Diagnosis and Treatment of Glaucoma and Neuroophthalmology, Eye Clinic, Department of Neurological Science, Ophthalmology, Genetics, University of Genoa, 16132 Genoa, Italy

## Abstract

*Purpose*. To determine whether the patterns of visual field damage between high-tension glaucoma (HTG) and normal-tension glaucoma (NTG) are equivalent. *Methods*. In this retrospective cross-sectional study, fifty-one NTG and 57 HTG patients were recruited. For each recruited patient only the left eye was chosen. Glaucomatous patients had abnormal visual fields and/or glaucomatous changes at the optic nerve head. They were classified as HTG or NTG on the basis of intraocular pressure (IOP) measurements. Patients' visual fields were analyzed by using Humphrey Field Analyzer (HFA), program 30-2, full threshold. The visual field sensitivity values and the pattern deviation map values of the 72 tested points were considered. Then a pointwise analysis and an area analysis, based on the Glaucoma Hemifield test criteria, were performed, and a comparison between the two subgroups was made by Student's *t* test. *Results*. Between NTG and HTG, no significant difference was found pointwise for almost all the visual field points, except for two locations. One was under the blind spot, and the other was in the inferior hemifield around the twenty-degree position. When area analysis was considered, three areas showed a significantly different sensitivity between HTG and NTG. *Conclusions*. These data suggested that there was no relevant difference in the pointwise analysis between NTG and HTG; however, when visual field areas were compared, no difference in paracentral areas was found between NTG and HTG, but superior nasal step and inferior and superior scotomata showed to be deeper in HTG than in NTG.

## 1. Introduction

Primary open angle glaucoma (POAG) could be easily divided into two subgroups based on the intraocular pressure (IOP) value. Some authors have noted optic disc and visual field differences between patients with high-tension glaucoma (HTG) and normal-tension glaucoma (NTG) [1–13]. In particular, the visual field damage in NTG was more likely to be dense, localized, and closer to fixation [4] while the optic disc appearance was characterized by larger optic discs, thinner infero-temporal rim areas, more pallor than cupping, and a pale, sloping, and moth-eaten appearance [2]. Other authors believed that the appearance of the optic disc and visual field in patients with NTG was similar to that found in HTG [14–18]. Furthermore, using a confocal scanning laser ophthalmoscope, Iester and Mikelberg did not find any morphometrical difference between NTG and HTG subgroups [19].

It was possible that different visual field locations of the glaucoma damage could have equivalent visual field index values; thus, similar visual field indices did not mean that the glaucomatous damage was in the same areas of the visual field. For this reason, in this study, we compared pointwise the NTG and HTG visual field of recruited patients to better evaluate the damage position. Then, to avoid loss of spatial information, the visual field maps were divided into ten different areas and compared between NTG and HTG.

## 2. Patients and Methods

This was a retrospective cross-sectional study. The research followed the tenets of the Declaration of Helsinki and was approved by the UBC-institutional review board. 

Three-hundred five consecutive clinical files of glaucoma patients were revised in one year (1996). All the examined clinical files were from the patients who attended at the glaucoma center of the Eye Care Centre, UBC. Patients were not excluded on the basis of gender, age, or race. In the present study, no patient had a refractive error greater than ±7 diopters (spherical equivalent). Patients with ocular/systemic disease potentially associated with optic neuropathy were excluded (i.e., anterior ischaemic optic neuropathy and hemodynamic crises). Visual acuity had to be better than 20/40 in all patients.

Patients were defined as having POAG if they had an abnormal visual field (as described below) and/or an abnormal ONH/retinal nerve fiber layer (RNFL) using a Volk 90° lens, an open angle by gonioscopy, and no clinically apparent secondary cause for their glaucoma.

The abnormal ONH and RNFL classification was based on the presence of a optic rim notch or diffuse/generalized loss of optic rim tissue, vertical cup/disc diameter ratio asymmetry unexplained by size differences in optic discs, disc hemorrhage, or a localized defect within the RNFL. The glaucomatous ONH damage had to be consistent with the patient's visual field defect.

The visual fields were assessed by Humphrey Field Analyzer (HFA, Humphrey Instruments, San Leandro, CA, USA). All patients were experienced in perimetry as they had already done at least three tests in the last three years.

The visual fields of all the subjects were analyzed by HFA, program 30-2. Subjects were classified as having an abnormal visual field if they had at least (a) 3 adjacent points depressed by 5 dB with one of the points being down by at least 10 dB, (b) 2 adjacent points down by 10 dB, or (c) a 10 dB difference across the nasal horizontal meridian in 2 adjacent points, all verified on at least three visual fields. None of the points could be edge points except immediately above or below the nasal horizontal meridian [20, 21]. Only reliable fields were used as determined by the reliability parameters (false positive responses and false negative responses <30% and fixation losses <10%). Mean deviation, pattern standard deviation, and corrected pattern standard deviation were used to characterize results in the two POAG subgroups. From all visual field maps, the sensitivity of each tested point and the pattern deviation map values of each point were analyzed.

From the clinic POAG files, NTG patients were chosen when IOP < 21 mmHg after a diurnal tension curve was taken every 2 hours without any topical or systemic treatment. Furthermore, no history of high IOP (greater than 21 mmHg) was present [22]. Then an age and refraction matched HTG subgroup was selected, and all patients had to have IOP > 21 mmHg without any treatment in at least three measurements done in different weeks. Among the latter subgroup, a visual field index matched group was selected to better compare patients with the same stage of the disease to NTG.

### 2.1. Statistical Analysis

For each patient only the left eyes were included in this study. The data of the two glaucomatous subgroups were analyzed by a descriptive analysis.

Visual field maps were compared pointwise by using sensitivity map values and pattern deviation map values between HTG and NTG. Then to avoid loosing spatial information, we analyzed the same data dividing the visual field maps into ten different areas using the HFA Glaucoma Hemifield Test (GHT) criteria. The values of the 10 GH areas were calculated by using the sensitivity and pattern deviation map values and compared between HTG and NTG.

Student's *t* test was used to compare data between HTG and NTG subgroups when the distribution of the data was normal. The Mann-Whitney nonparametric test was used instead when the distributions of the two subgroups data were nonnormal. A *P* value < 0.05 was considered to be statistically significant.

## 3. Results

From 305 clinical files, 51 consecutive NTG patients were recruited, and an age, refractive error, and visual field index values matched HTG group was created. 57 HTG eyes were included in the latter subgroup.

The mean age ± standard deviation (SD) of patients with HTG was 64.8 ± 12.1 years while patients with NTG had a mean age of 61.3 ± 13.2 years. This difference was not statistically significant. No significant difference was found between HTG and NTG for refractive error or for visual field indices (Table 1).

**Figure 1:**
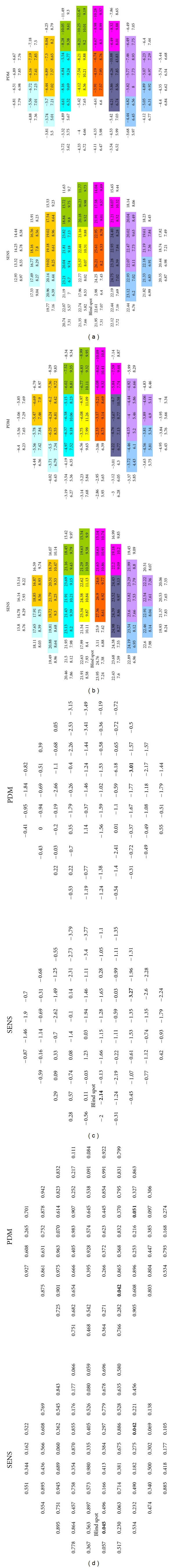
(a) Sensitivity (SENS) and pattern deviation map (PDM) values (mean and standard deviation (SD)) for each point of the visual field in HTG together with the 10 different glaucoma hemifield test (GHT) areas representation. (b) Sensitivity (SENS) and pattern deviation map (PDM) values (mean and standard deviation (SD)) for each point of the visual field in NTG together with the 10 different GHT areas representation. The different colours represent the different areas used by the GHT. (c) Difference of the sensitivity (SENS) values and the pattern deviation map (PDM) values pointwise between HTG and NTG. (d) Comparison (*P* values) of the difference pointwise of the sensitivity (SENS) values and the pattern deviation map (PDM) values between HTG and NTG. Bolding indicates a significant difference between the two subgroups.

When the sensitivity maps were analyzed pointwise, two significant points were found to be statistically different (Figure 1). One was under the blind spot, and the other was in the inferior hemifield around the twenty degree isopter. In both locations, the mean sensitivity of NTG subgroup was higher than in the HTG one. Furthermore, in the sensitivity perimetric map, there was a trend to be significant in seven other visual field points, and in all these points HTG visual field was worse than those in NTG. Three points of these were on the superior nasal area creating a possible cluster. Of the 72 analyzed points, in 12 locations, the result of the difference between HTG and NTG sensitivity was positive while in 64 points the data were negative (Figure 1(c)). 

When the pattern deviation maps were analyzed pointwise, two significant points were found to be statistically different (Figure 1). One was under the blind spot, another was in the inferior hemifield around the twenty degrees isopter. In these locations, the mean sensitivity of NTG subgroup was higher than in the HTG one. Furthermore, in the pattern deviation map, there was a trend to be significant in other two visual field points. Of the 72 analyzed points, in 65 locations, the result of the difference between HTG and NTG sensitivity was negative (Figure 1(c)). 

**Figure 2:**
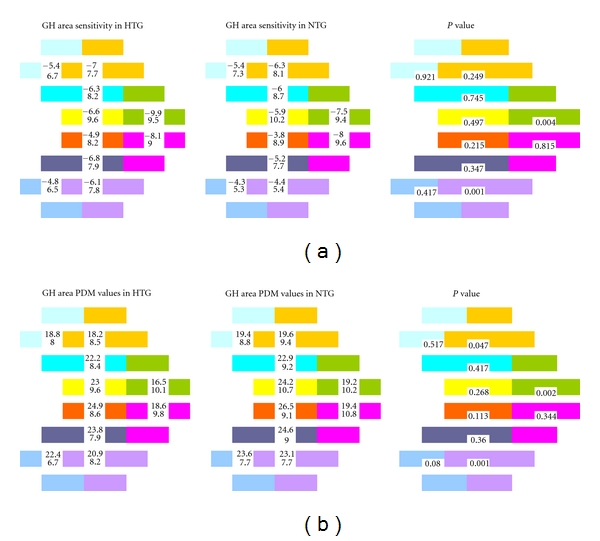
(a) Comparison (*P* value) of the sensitivity values (mean and standard deviation) of the 10 glaucoma hemifield test (GHT) areas between HTG and NTG. The different colours represent the different areas used by the GHT. (b) Comparison (*P* value) of the pattern deviation map (PDM) values (mean and standard deviation) of the 10 GHT areas between HTG and NTG. The different colours represent the different areas used by the GHT.

When the 10 different GHT areas were compared between HTG and NTG by using the sensitivity value map, two areas were significantly different (Figure 2(a)). One was in the inferior hemifield around the twenty degree isopter, and the other was on the superior nasal step. But when we used the pattern deviation value map, three areas were significantly different between NTG and HTG (Figure 2(b)). One was in the inferior hemifield around the twenty degrees isopter, the next was in the superior hemifield around the twenty degrees isopter, and the last one was on the superior nasal step. 

## 4. Discussion 

There remains considerable disagreement within the glaucoma community as to the possible differences in optic disc appearance and visual field damage present in patients with HTG and NTG [1–18]. Caprioli and Spaeth showed that scotomas in NTG had a steeper slope and were significantly closer to fixation compared to HTG and with a greater depth [4]. Greve and Geijssen detected differences in the distribution of the visual field defects between HTG and NTG. In the latter, the larger defects were more frequently in the upper half of the visual field [23]. However many years earlier, both Bjerrum and later Sjogren did not find any difference between these two subgroups [4, 5]. Also Drance did not find any differences in the characteristics of the visual field of HTG, NTG, and ischaemic anterior optic neuropathy with Goldmann perimetry [24]. Many other studies were possible to find in the literature, and some authors believed that HTG and NTG had different visual field defects and ONH damage [1–13] while others found that the optic disc and visual field appearances were similar between the two subgroups [14–19]. 

These different findings might be related to a selection bias present since NTG was usually detected only when significant ONH damage had already occurred, or significant visual field impairment was present. Furthermore, in cases where visual field defects were related to subjective visual impairment, it might be more evident to patients with NTG to attend their physicians only if the scotomata were close to fixation. In HTG, patients were mainly detected by objective high IOP and not by the position of scotomata. This could be one reason for the different variations of scotomata between HTG and NTG found in the literature. In this study, 72 points of the visual field were considered, but, in most of the single tested points, no significant difference was found both in the sensitivity map and in the pattern deviation map. 

Although no statistical point-wise difference was found between NTG and HTG for most of the tested points, we had to point out two statistical considerations. First, when so many numbers (72 points) were tested and compared, for chance it was possible to find some points with significant difference just because of mathematical probability (Figures 1(c) and 1(d)). Second, if we applied the binomial (sign) test to the pattern of the mean changes across the points and the test assumed that point data were mutually independent, under the hypothesis of zero difference, the positive and negative point data are equally likely. In Figure  2, it has been shown the difference between HTG and NTG for each point in the sensitivity map (a) and in the pattern deviation map (b) and we found 64 and 65 negative points on 72, respectively. This means that HTG defect had a deeper visual field damage than NTG, although MD, PSD, and CPSD did not show any significant difference (Table 1). When the GHT areas were compared between HTG and NTG, some difference was found, in the superior nasal step and inferior arcuate scotomata areas; however, no significance difference was found in the two paracentral areas. 

Also Araie et al. analyzed the visual field pointwise and found different visual field morphology between NTG and HTG. They suggested that different ONH regions could be more susceptible to damage in NTG [25, 26]. Our sample was different from the latter because the MD was about −7 ± 5 dB in both subgroups, and this could be one of the reason for different results. However, in our sample, patients with HTG had worse MD by as much as 1.3 dB than NTG patients, but the difference was not significant. It could be also possible that greater difference in IOP could also be likely to show a difference between the two groups. 

In this study as well as in most of the previous mentioned ones [1–17, 23–26], central corneal thickness was not measured. We divided the POAG group into two subgroups (NTG and HTG subgroup) based on IOP measurements to better compare our results to those of previous studies [1–17, 23–26]. The recent knowledge of the importance of cornea thickness in determining accurate IOP values made the boundary between the two diseases unclear. Furthermore, using these data, it would be possible to understand how IOP values were not enough to separate different POAG subgroups. HTG could have POAG diagnosed because of a thick cornea while NTG could be defined as POAG due to a thin cornea. Clinically, even if corneal thickness could be assessed in most of the patients, it has been suggested by the European Glaucoma Society that corneal thickness should be determined in NTG or HTG or ocular hypertension only when findings do not match [22]. However, it is not completely understood its role in determining the real IOP values and glaucoma progression [27]. 

Many other risk factors are involved in the pathogenesis of glaucoma such as low blood pressure, migraines, repeated disc hemorrhages, gender, and vasospastic phenomenon, but, until now, IOP is still the main parameter to classify HTG and NTG [22]. A patient with migraine, repeated disc hemorrhages, and a different approach to classify POAG could help to better classify patients. For instance, several authors suggested to observe the ONH morphology [28, 29]. If these data from functional analysis and those from structure analysis [19] were considered together, we might presume that NTG and HTG could be the same disease. However, in the group of POAG, different risk factors could be related to different modes of development of damage [28, 29]. 

In conclusion, these data suggested that there was no relevant difference in the pointwise analysis between NTG and HTG; however, when visual field areas were compared, no difference in paracentral areas was found between NTG and HTG, but superior nasal step and inferior and superior scotomata areas showed to be deeper in HTG than in NTG. 

## Figures and Tables

**Table 1:** Descriptive analysis in high-tension glaucoma (HTG) and normal-tension glaucoma (NTG).

	NTG		HTG		*t* test
	(*n* = 51)		(*n* = 57)		
	mean	SD	mean	SD	*P *value
Age (years)	61.3	13.2	64.8	12.1	0.261
Refractive error (diopters)	1.96	2.96	1.88	3.58	0.932
Mean deviation (dB)	−6.31	6.01	−7.69	5.02	0.265
Pattern standard deviation (dB)	7.08	4.16	7.52	3.38	0.611
Corrected pattern standard deviation (dB^2^)	6,51	4.3	6.97	3.44	0.593

*n*: number of eye considered, SD: standard deviation.
